# Effects of Supplementing Yeast Culture to Prepartum Cows Under Heat Stress on the Jejunal Microbiota and Metabolites of Calves

**DOI:** 10.3390/ani16040668

**Published:** 2026-02-20

**Authors:** Bosen Zhang, Ziye Zhang, Lei Feng, Zhiyong Hu, Ruina Zhai

**Affiliations:** Key Laboratory of Efficient Utilization of Non-Grain Feed Resources (Co-Construction by Ministry and Province), Ministry of Agriculture and Rural Affairs, Shandong Provincial Key Laboratory of Animal Nutrition and Efficient Feeding, College of Animal Science and Technology, Shandong Agricultural University, Tai’an 271017, China; zbs1123510270@163.com (B.Z.); ziye0322@163.com (Z.Z.); flei0921@163.com (L.F.); hzy20040111@126.com (Z.H.)

**Keywords:** heat stress, yeast culture, dairy calves, gut microbiota

## Abstract

Heat stress, particularly during the prepartum period, compromises dairy cow health and has lasting effects on offspring. This study aimed to investigate the effects of maternal yeast culture (YC) supplementation during heat stress on the intestinal health of newborn calves, with a specific focus on elucidating the associated changes in jejunal morphology, microbial community, and metabolic profiles. Cows receiving YC had lower rectal temperatures, confirming mitigated heat stress. Their calves showed enhanced immune status (higher IgA, IgG) and reduced oxidative stress and metabolic markers. Jejunal structure and barrier integrity were improved. While microbial diversity was similar, specific bacterial abundances differed (e.g., *Enterococcus* spp. enriched in YC calves). Metabolomic analysis revealed modulated bile acid pathways and metabolites in YC calves, correlating with the microbial shifts. In conclusion, maternal YC supplementation during heat stress supports calf early-life gut health and development through maternal-offspring programming, optimizing gut microbiota, metabolism, and immunity.

## Simple Summary

Heat stress during late pregnancy negatively impacts dairy cow welfare and fetal development. This study demonstrates that supplementing heat-stressed cows with yeast culture before calving significantly improves the health of their offspring. Newborn calves from supplemented dams exhibited stronger immune systems, reduced oxidative stress, and better intestinal development. These physical benefits were linked to a healthier gut microbiome and improved metabolic balance. Consequently, maternal yeast culture supplementation offers a practical nutritional strategy to mitigate the damaging effects of heat stress on the next generation, promoting better calf survival, growth, and economic sustainability for dairy producers.

## 1. Introduction

Heat stress is a major environmental constraint on the sustainability of dairy production [1]. Dairy cows begin to exhibit heat-stress responses when the temperature–humidity index (THI) exceeds 72 [2]. Notably, when heat stress coincides with prepartum stress, the combined challenge can synergistically increase the risk of maternal metabolic disorders and exert long-term effects on offspring development [3]. Heat stress induces systemic inflammation in the dam and increases circulating concentrations of inflammatory mediators and metabolites [4,5]. These factors may act on the rapidly developing fetus through the placenta, thereby disrupting normal intestinal structural and functional maturation [6]. Developmental programming, also known as fetal programming, refers to the concept that stress factors during development can alter the gene expression in the fetus or newborn through epigenetic mechanisms [7,8]. In the context of the fetal intestine, such programming may manifest as impaired villus development, altered villus morphology, and downregulation of tight junction–related gene expression. Collectively, these changes may increase intestinal permeability and the risk of “leaky gut” in newborn calves [9,10,11]. In addition, an inflammatory intrauterine environment may prematurely activate the fetal immune system and promote a proinflammatory phenotype [12]. Heat stress can also disrupt the maternal microbiota and may influence early-life microbial colonization in offspring through the gut–mammary axis [13], potentially impairing the establishment of a healthy microbial community [14] and further challenging intestinal homeostasis in calves.

The jejunum is a core segment of the small intestine and the primary site of nutrient absorption; thus, the quality of early jejunal development is closely associated with neonatal survival and growth potential. During fetal life, intestinal morphogenesis is largely completed, and by late gestation, the jejunal mucosa exhibits a typical villus–crypt architecture, with continued villus elongation in preparation for the high nutrient load from milk after birth. Physiologically, newborn calves resemble monogastric animals, and efficient nutrient utilization relies heavily on jejunal digestive enzyme activity and the expression of nutrient transporters [15]. Moreover, the intestine is not only a digestive organ but also the largest immune organ. In early life, the mucosal immune system undergoes rapid development and functional maturation [16]. Therefore, optimizing the jejunal developmental environment through early-life nutritional and management strategies is critical to ensure absorptive capacity and immune function in newborn calves.

Yeast culture (YC) is a concentrated, dried product derived from high-density liquid fermentation followed by deep solid-state fermentation using saccharomyces cerevisiae as the microbial strain. It contains yeast metabolites, yeast cells and a denatured culture medium, and has been shown to significantly enhance immunity, alleviate stress, and improve productivity [17]. It contains a broad range of bioactive components, including beta-glucans, mannan oligosaccharides, nucleotides, peptides, B vitamins and various organic acids and metabolites [18]. YC has been reported to improve ruminal fermentation, fiber digestion, and microbial protein synthesis, and to enhance intestinal barrier function, modulate immune responses, and alleviate oxidative stress [19,20]. Previous studies have shown that YC supplementation can mitigate heat stress in dairy cows, as evidenced by increased dry matter intake, reduced respiratory rate, and improved postpartum health status. Specifically, YC has been associated with lower circulating concentrations of nonesterified fatty acids (NEFAs) and beta-hydroxybutyrate (BHBA) as well as reduced incidences of metritis and mastitis, thereby improving lactation performance [21,22,23]. In heat-stressed beef cattle, YC has also been shown to optimize ruminal fermentation (e.g., increased total volatile fatty acids and improved fiber digestibility) and enhance systemic antioxidant capacity, ultimately improving average daily gain, feed efficiency, and carcass traits [24]. However, most existing studies have focused on the direct effects of YC on performance and health in heat-stressed dams. Whether maternal YC supplementation during the critical prepartum window can confer protective, programmatic benefits to the offspring—specifically targeting the foundational development of the small intestine—remains unclear. In ruminants, the basic structure of the small intestine is formed by the middle of pregnancy, but the final stage of functional maturity, including the rapid elongation of villi and the development of crypts, mainly occurs in the last three months of pregnancy, especially in the weeks before delivery. During this period, the fetal intestine is particularly sensitive to maternal signals (including nutrients and inflammatory factors) [25,26]. During this period, the fetal intestine is particularly sensitive to maternal signals (including nutrients and inflammatory factors) [27]. Studies have shown that feeding rumen lysine to cows 3 weeks before delivery may increase amino acid transport mediated by the placenta to the fetus and enhance protein synthesis in calves [28]. Elolimy et al. added rumen-protected methionine to cows 4 weeks before delivery, causing the microbial and metabolic profiles of the offspring to shift towards a more efficient phenotype [29]. Therefore, intervention during the late pregnancy window is strategically significant, as it can directly regulate the maternal environment, thereby influencing the critical late stage of fetal jejunum development and the early colonization of microorganisms, which will have a lasting impact on the health after birth.

To address this knowledge gap, this study aimed to evaluate the intergenerational impact of maternal YC supplementation during prepartum heat stress on neonatal calf intestinal health. We hypothesized that supplementing YC to heat-stressed prepartum cows would program neonatal calf jejunal development, leading to coordinated improvements in intestinal morphology, beneficial shifts in the early-life microbiota, and a more homeostatic metabolic microenvironment.

## 2. Materials and Methods

### 2.1. Ethical Statement

All animal procedures, including euthanasia and tissue collection, were approved by the Animal Welfare and Experimental Ethics Committee of Shandong Agricultural University (approval number: SDAUA-2024-232). The experiment was conducted from July to September 2024 on a commercial dairy farm in Shandong Province, China.

### 2.2. Animals and Experimental Design

A randomized matched-pair design was utilized. Twenty-four heat-stressed Holstein cows in late gestation (21 d before expected calving), matched for parity (parity 2) and body condition score (BCS = 3.0–3.5), were enrolled and randomly assigned to 2 groups: PCON (*n* = 12), fed a basal diet; and PYC (*n* = 12), fed the basal diet supplemented with 30 g/d of yeast culture from d −21 until calving. The daily YC supplement for each cow was individually weighed and top-dressed onto a small portion of the morning TMR to ensure complete consumption before offering the remainder of the daily feed. Saccharomyces cerevisiae culture “Baihuibang 4C” were provided by Beijing Enhalor International Tech Co., Ltd. (Beijing, China). Per kilogram of yeast culture contains ≥20% crude protein, ≤11% crude ash, ≥2.0% mannan, and ≤10% moisture. At the start of the supplementation period (day −21), the 24 cows were randomly assigned to the PCON or PYC group. After calving, to control for sex-related variation, twelve male calves born to PCON dams and twelve male calves born to PYC dams were enrolled as the CON (*n* = 12) and YC (*n* = 12) groups, respectively, for all subsequent investigations. This approach was taken to eliminate sex as a source of variation, thereby increasing the sensitivity to detect the specific effects of maternal YC supplementation.

**Figure 1:**
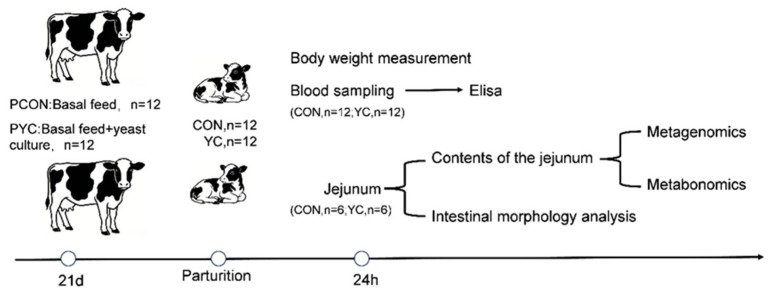
Experimental design. PCON = heat-stressed prepartum cows fed the basal diet; PYC = heat-stressed prepartum cows fed the basal diet plus yeast culture; CON = calves born to PCON dams; YC = calves born to PYC dams.

Twelve male calves born to dams in each group were enrolled: CON (*n* = 12), born to PCON dams; and YC (*n* = 12), born to PYC dams. After birth, the calves were separated from their mothers and were prohibited from drinking water and receiving colostrum before sampling. Blood samples were collected from all 24 calves immediately after birth. Subsequently, 12 calves (6 per group) were randomly selected for euthanasia and jejunal sampling (Figure 1).

Prepartum cows were housed in loose pens and fed a total mixed ration (TMR) formulated to meet nutrient requirements (NY/T34-2004). The formulation of the basal diet and its nutrient composition are provided in Appendix A (Table A1). Cows had ad libitum access to feed and water, with fresh feed delivered 3 times daily at 08:00, 14:00, and 19:30.

### 2.3. Data and Sample Collection

#### 2.3.1. Temperature-Humidity Index and Rectal Temperature

Ambient temperature (°C) and relative humidity (%) were recorded daily at 07:00, 14:00, and 22:00 using an electronic thermometer–hygrometer (Model: COS-04 Shandong Renke Measurement & Control Technology Co., Ltd., Jinan, China) positioned at cow head height. The temperature–humidity index (THI) was calculated according to Thom (1959) [30]:

THI = (0.8 × T) + [(RH/100) × (T − 14.3)] + 46.4

where T denotes the cowshed temperature (°C); RH denotes the relative humidity of the cowshed (%).

Rectal temperature was measured weekly from d −21 until calving. Measurements were taken between 09:30 and 10:30 by the same 2 technicians using a veterinary thermometer.

#### 2.3.2. Jejunal Tissue and Digesta Collection

Selected calves were euthanized via intravenous injection of 4% sodium pentobarbital (40 mg/Kg of BW). To minimize postnatal microbial exposure, all selected calves were euthanized and sampled within 10–15 min after birth, before standing, suckling, or extended contact with the dam or environment. During handling, oral contact with the dam’s vagina, body, or bedding was prevented. This rapid protocol aimed to capture intestinal microbiota and metabolome profiles prior to substantial extra-uterine influence, thus reflecting in utero programming effects. Following confirmation of euthanasia, calves were exsanguinated. The gastrointestinal tract was immediately removed, and a 20 cm segment of the mid-jejunum was isolated and clamped with hemostatic forceps. Digesta and tissue samples were collected into cryovials, snap-frozen in liquid nitrogen, and stored at −80 °C. Additional tissue samples were rinsed with phosphate-buffered saline (PBS) and fixed in 4% paraformaldehyde for histological analysis.

#### 2.3.3. Histology and Morphometric Analysis

Jejunum tissue samples were fixed in 4% paraformaldehyde for at least 72 h. The samples were then dehydrated through a series of ethanol concentrations (50%, 70%, 80%, 90%, and 100%), cleared in xylene, and embedded in paraffin blocks in the vertical direction. Four-micrometer-thick sections were cut from each paraffin block using a LEICA RM2235 microtome. The sections were stained with hematoxylin and eosin (H&E) according to the manufacturer’s instructions (Leica Microsystems GmbH, Simi Valley, CA, USA) and observed under an optical microscope at a magnification of 10×. Villus height (VH) and crypt depth (CD) were measured using ImageJ software (version 1.54f, National Institutes of Health, Bethesda, MD, USA). A total of 30 well-oriented villus–crypt units were quantified per group, and the ratio of villus height to crypt depth (VH:CD) was calculated.

#### 2.3.4. Blood Sampling and Serum Analysis

Blood was collected via jugular venipuncture immediately after birth into sterile, 10 mL evacuated serum tubes (Model: YL021, Jiangsu Yuli Medical Instrument Co., Ltd., Jiangsu Taizhou, China) containing no anticoagulant. Samples were centrifuged at 3000× *g* for 15 min at 4 °C to harvest serum using a benchtop low-speed centrifuge (model L2-6K, Shanghai Zhaodi Biotechnology Co., Ltd., Shanghai, China). Serum was aliquoted into 2 mL microcentrifuge tubes and stored in liquid nitrogen until analysis. Serum concentrations of IgA, IgG, IL-6, BHBA, MDA, NEFA, and cortisol were determined using ELISA kits (Nanjing Jiancheng Bioengineering Institute, Nanjing, China) according to the manufacturer’s instructions. Specifically, bovine IgA and IgG concentrations were measured using the respective kits (Catalog numbers: [H108-1-1] and [H106-1-1]), which employ antibodies specific to bovine immunoglobulins.

#### 2.3.5. DNA Extraction, Metagenomic Sequencing, and Bioinformatics Analysis

Total genomic DNA was extracted from jejunal digesta samples using the Magen Pure Soil DNA Kit (Guangzhou, China) according to the manufacturer’s instructions. DNA quantity and quality were assessed using a Qubit fluorometer and a NanoDrop spectrophotometer (Thermo Fisher Scientific, Waltham, MA, USA). Qualified genomic DNA was sheared to an average fragment size of ~350 bp, followed by end repair and A-tailing. Illumina sequencing adapters were ligated using the NEBNext^®^ Ultra™ DNA Library Prep Kit (NEB, Ipswich, MA, USA). Fragments of 300–400 bp were PCR-amplified to enrich the library. PCR products were purified using the AMPure XP system (Beckman Coulter, Brea, CA, USA). Library size distribution was evaluated using an Agilent 2100 Bioanalyzer (Agilent Technologies, Böblingen, Germany), and library concentration was determined by quantitative real-time PCR. Libraries were sequenced on a NovaSeq X Plus platform using a paired-end 150 bp strategy (PE150). Raw reads were quality-filtered using fastp (v0.20.0) [31]. Clean reads from each sample were assembled de novo using MEGAHIT (v1.2.9) [32], and contigs ≥500 bp were retained. Gene prediction was performed on assembled contigs using MetaGeneMark (v3.38). Predicted genes were clustered using CD-HIT (http://www.bioinformatics.org/cd-hit/,v4.6, accessed on 12 September 2025), in which sequences with ≥95% identity and >90% read coverage were merged into a single cluster; the longest sequence in each cluster was selected as the representative sequence to construct a nonredundant gene catalog [33,34]. Clean reads were mapped back to the representative gene catalog using Bowtie2 (v2.3.5.1) to obtain read counts. Pathoscope (v2.0.7) was used to reassign reads to the most likely source genes, and genes supported by fewer than 2 reads were removed to generate the final nonredundant gene set [35,36]. Taxonomic annotation and relative abundance profiles were generated by aligning the nonredundant gene set to the NCBI NR database and applying the lowest common ancestor (LCA) algorithm implemented in MEGAN (v6.25.10) [37,38]. Functional annotation was conducted using DIAMOND ((http://ab.inf.uni-tuebingen.de/software/diamond/, version 2.0.11, accessed on 12 September 2025); e-value < 1 × 10^−5^) against the KEGG database (Release 111) and the CAZy database (v2024.07.14) [39].

#### 2.3.6. Metabolomic Analysis of Jejunal Digesta

Approximately 100 mg of each jejunal digesta sample was weighed and extracted with 1 mL of prechilled methanol:acetonitrile:water (2:2:1, *v*/*v*/*v*). Samples were homogenized using an MP homogenizer (24 × 2; 6.0 m/s; 20 s; 3 cycles), followed by low-temperature ultrasonication for 30 min per cycle (2 cycles). The extracts were incubated at −20 °C for 60 min and then centrifuged at 13,000× *g* for 15 min at 4 °C. The supernatant was collected (aliquoted at 900 μL per tube), vacuum-dried, and stored as a lyophilized powder at −80 °C until analysis. For LC–MS analysis, dried extracts were reconstituted with 100 μL of acetonitrile:water (1:1, *v*/*v*), vortexed, and centrifuged at 14,000× *g* for 15 min at 4 °C. The resulting supernatant was transferred for instrumental analysis. Metabolites were separated using an Agilent 1290 Infinity UHPLC system equipped with a HILIC column. Samples were maintained in an autosampler at 4 °C throughout the analytical run and were analyzed in a randomized order to minimize potential bias from instrument signal drift. MS1 and MS2 data were acquired in both positive and negative ionization modes using an AB TripleTOF 6600 mass spectrometer. Raw data files were converted to mzML format using ProteoWizard (v3.0.6428). Peak alignment, retention-time correction, and peak area extraction were performed using XCMS (Online v3.7.1). The resulting data were used for metabolite identification, preprocessing, and downstream statistical analyses. Metabolite traceability and enrichment analyses were conducted using MetOrigin (http://metorigin.met-bioinformatics.cn/app/metorigin, accessed on 2 October 2025).

### 2.4. Statistical Analysis

GraphPad Prism 10 (GraphPad Software, San Diego, CA, USA) was used to analyze rectal temperature, serum variables, and jejunal villus morphology. Differences between 2 groups were evaluated using a two-tailed unpaired Student’s *t* test. Statistical significance was indicated as follows: *p* < 0.05 (*), *p* < 0.01 (**), *p* < 0.001 (***), and *p* < 0.0001 (****).

For microbiome analyses, α-diversity indices (Shannon and Chao1) and differential taxa between groups were compared using the Wilcoxon rank-sum test. Bar plots were generated in GraphPad Prism 10, and *p* < 0.05 was considered statistically significant. Beta-diversity was calculated based on Bray–Curtis distances and assessed using analysis of similarity (ANOSIM).

Differential functional profiles of the microbiome were analyzed using Welch’s *t* test based on KEGG and CAZyme annotations, with *p* < 0.05 considered significant.

Differential metabolites were identified using orthogonal partial least squares discriminant analysis (OPLS-DA) combined with *t* tests. Metabolites were considered differential when the false discovery rate (FDR)–adjusted *p* value was <0.05 and the variable importance in projection (VIP) score was >1.

All correlation analyses were performed using Spearman’s rank correlation, and associations were considered meaningful when |*r*| > 0.50 and *p* < 0.05. Multi-omics networks were visualized using Gephi (v0.10).

## 3. Results

### 3.1. Environmental Conditions and Maternal Physiological Response

**Figure 2:**
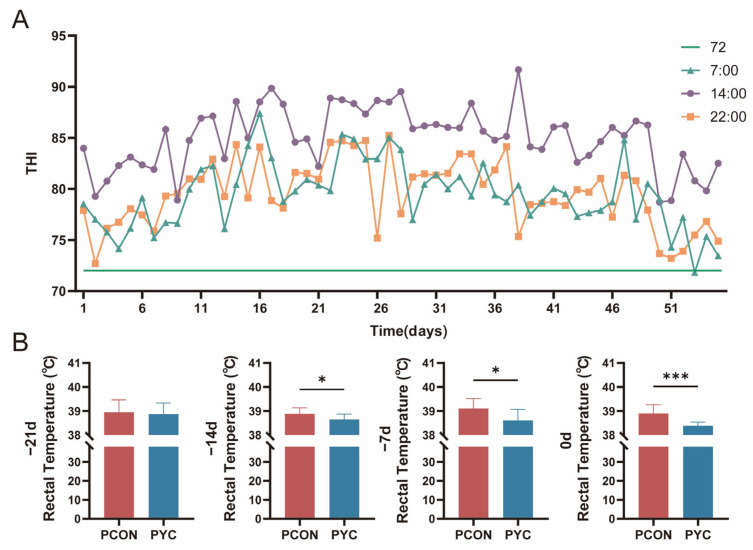
(**A**) Temporal changes in the temperature–humidity index (THI) in the barn during the experimental period. (**B**) Effects of yeast culture supplementation on rectal temperature in heat-stressed prepartum cows. PCON = heat-stressed prepartum cows fed the basal diet (*n* = 12); PYC = heat-stressed prepartum cows fed the basal diet plus yeast culture (YC; *n* = 12). Day −21 indicates 21 d before calving (start of YC supplementation in PYC), day −14 indicates 14 d before calving, day −7 indicates 7 d before calving, and day 0 indicates the day of calving. *p* < 0.05 (*) and *p* < 0.001 (***).

Throughout the entire experiment, the thermal stress index (THI) of the cows remained above the threshold of 72 (Figure 2A), and the average temperature of the barn environment was at least 27.1 °C throughout the study period, which was always higher than 25.8 °C and exceeded the thermal neutral zone of the cows (Table A2). These data collectively indicate that the cows were always in a heat-stressed environment. Compared with the PCON group, after adding yeast, the rectal temperature of the dairy cows in the PYC group significantly decreased (*p* < 0.05; Figure 2B), indicating that yeast alleviated the elevated body temperature that occurred in prepartum cows due to heat stress.

### 3.2. Serum Indices and Jejunal Morphology

**Figure 3:**
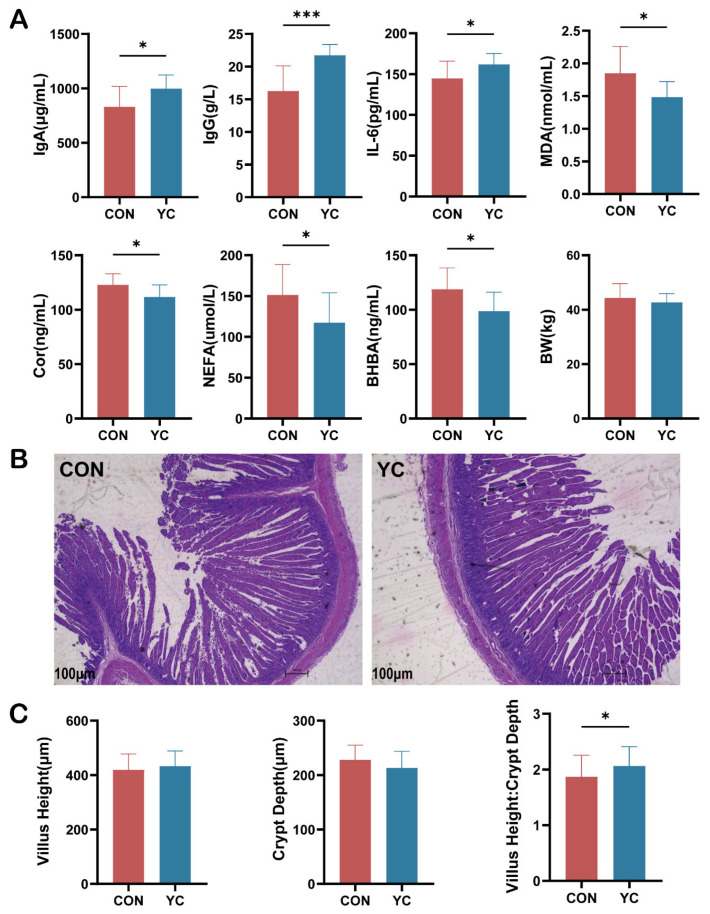
Effects of maternal yeast culture supplementation on (**A**) serum immune, metabolic, and oxidative stress–related variables in newborn calves, (**B**) jejunal histomorphology, and (**C**) villus height, crypt depth, and villus height-to-crypt depth ratio (VH:CD). CON = calves born to heat-stressed dams fed the basal diet ((**A**): *n* = 12; (**B**,**C**): *n* = 6); YC = calves born to heat-stressed dams fed the basal diet plus yeast culture ((**A**): *n* = 12; (**B**,**C**): *n* = 6). *p* < 0.05 (*) and *p* < 0.001 (***). IgA = Immunoglobulin A, IgG = Immunoglobulin G, IL-6 = Interleukin-6, MDA = Malondialdehyde, Cor = Cortisol, NEFA = Non-Esterified Fatty Acids, BHBA = β-Hydroxybutyric Acid, BW = Body Weight.

Maternal YC supplementation significantly altered serum profiles in newborn calves. Compared with CON calves, YC calves exhibited greater serum concentrations of IgA, IgG, and IL-6 (*p* < 0.05; Figure 3A). Conversely, concentrations of metabolic and stress indicators—including BHBA, NEFA, MDA, and cortisol—were lower in YC calves than in CON calves (*p* < 0.05). The increase in these immunoglobulins may indicate that the maternal YC intervention has facilitated the accelerated maturation of the fetal immune system. The differences in oxidative and metabolic stress indicators may indicate that YC alleviated the oxidative and metabolic burden transferred from the mother’s heat stress to the fetus.

Histological evaluation of jejunal sections (Figure 3B) showed pronounced pathological alterations in CON calves, including disorganized epithelial cell arrangement, villus breakage and sloughing, and the presence of detached epithelial cells in the lumen. In contrast, jejunal villi in YC calves appeared relatively intact. Occasional villus fragmentation was observed in YC calves; however, the fracture edges appeared clean and complete, suggesting that these changes were likely attributable to mechanical damage during sample preparation rather than tissue pathology.

Quantitative morphometric analysis of villus height (VH) and crypt depth (CD) is shown in Figure 3C. Compared with CON calves, YC calves showed a tendency toward increased VH and decreased CD; however, neither parameter alone differed significantly between groups (*p* > 0.05). Despite the lack of significance for VH and CD individually, the villus height-to-crypt depth ratio (VH:CD) was significantly higher in YC calves than in CON calves (*p* < 0.05; Figure 3C). This finding indicates that although the maternal YC intervention did not completely alter the overall structure of the calves’ intestines, it played a positive role in protecting the integrity of the intestinal barrier.

### 3.3. Compositional Profiles of the Jejunum Microbiome and Taxonomic Differences Between the CON and YC Calves

Shotgun metagenomic sequencing generated a total of 826,452,390 reads, with an average of 68,871,033 ± 1,857,286 reads per sample (mean ± SEM). After quality control and removal of host-derived reads, 808,700,694 reads remained (67,391,725 ± 1,791,213 reads per sample). De novo assembly produced 125,234 contigs, with 62,617 ± 1410 contigs per group on average (Table A3).

**Figure 4:**
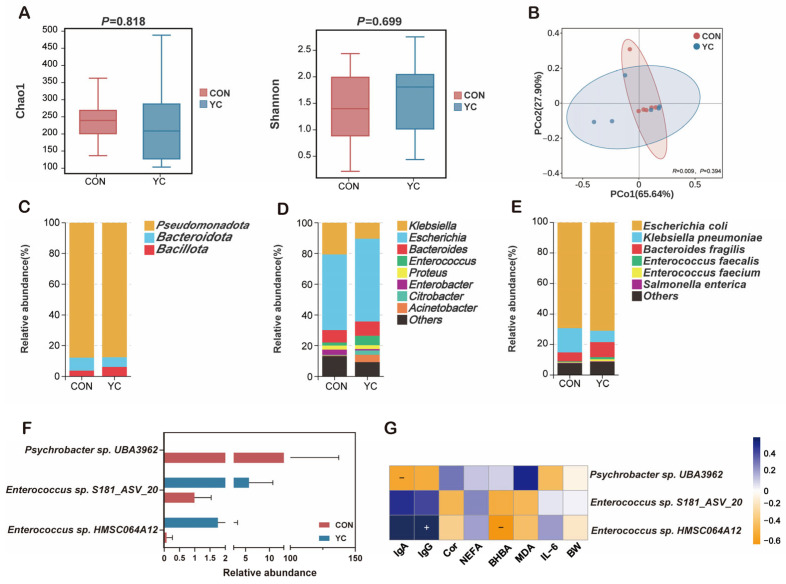
Differences in jejunal microbial diversity and community features between CON and YC calves. (**A**) Chao1 and Shannon indices. (**B**) PCoA based on Bray–Curtis distances with ANOSIM. (**C**) Phylum-level composition. (**D**) Genus-level composition. (**E**) Species-level composition. (**F**) Differential species identified by the Wilcoxon rank-sum test. (**G**) Spearman correlations between differential taxa and phenotypic variables.

Alpha-diversity analysis showed no differences in the Chao1 or Shannon indices between groups (*p* > 0.05; Figure 4A), indicating that maternal YC supplementation did not alter microbial richness or evenness in the calf jejunum. Principal coordinate analysis (PCoA) based on Bray–Curtis distances, together with ANOSIM, indicated no clear separation in overall community structure between groups (*R* = 0.009, *p* > 0.05; Figure 4B).

At the phylum level, *Pseudomonadota*, *Bacteroidota*, and *Bacillota* were the predominant taxa in both groups (Figure 4C). *Bacillota* tended to be higher in the YC group, whereas *Pseudomonadota* and *Bacteroidota* tended to be higher in the CON group; however, these differences were not statistically significant (*p* > 0.05). At the genus level, *Escherichia*, *Klebsiella*, *Bacteroides*, *Enterococcus*, *Acinetobacter*, *Proteus*, *Enterobacter*, and *Citrobacter* were dominant in both groups (Figure 4D). At the species level, *Escherichia coli*, *Klebsiella pneumoniae*, *Bacteroides fragilis*, *Enterococcus faecalis*, *Enterococcus faecium*, and *Salmonella enterica* were among the most abundant species detected in both groups (Figure 4E).

To identify potential group-associated taxa, we performed an exploratory species-level differential abundance analysis using the Wilcoxon rank-sum test. *Enterococcus* sp. *HMSC064A12* and *Enterococcus* sp. *S181_ASV_20* were enriched in the YC group, whereas *Psychrobacter* sp. *UBA3962* showed significantly higher relative abundance in the CON group (*p* < 0.05; Figure 4F). To investigate potential microbiota–host interactions, differential taxa were correlated with phenotypic variables using Spearman’s rank correlation (*p* < 0.05, *R* > 0.5). *Enterococcus* sp. *HMSC064A12* was positively correlated with serum IgG and negatively correlated with serum BHBA, whereas *Psychrobacter* sp. *UBA3962* was negatively correlated with serum IgA (Figure 4G).

### 3.4. Microbial Co-Occurrence Networks

**Figure 5:**
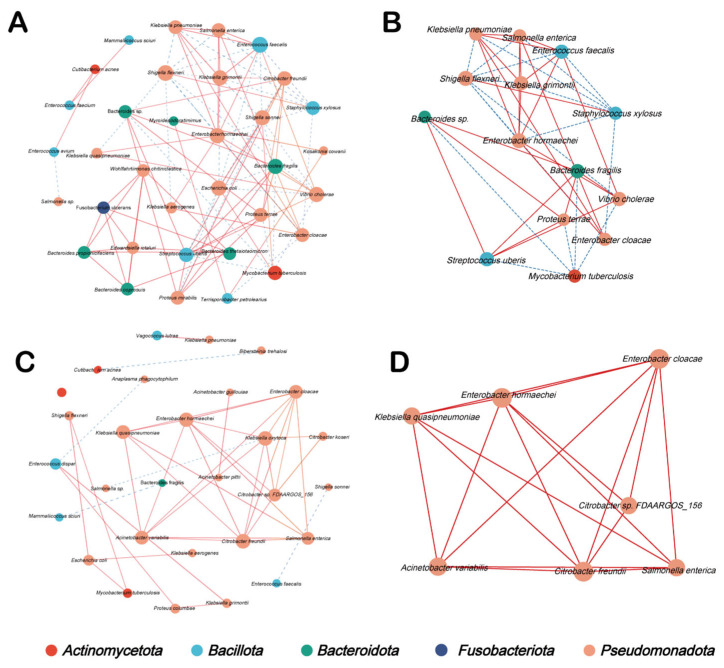
Co-occurrence networks of the jejunal microbiome. (**A**) Co-occurrence network in CON calves. (**B**) Core subnetwork in CON calves. (**C**) Co-occurrence network in YC calves. (**D**) Core subnetwork in YC calves. Edge colors indicate positive (red) or negative (blue) correlations between nodes.

Microbial co-occurrence analysis revealed distinct interaction patterns between groups. The CON group displayed a more complex global network structure, characterized by a greater number of nodes and edges compared with the YC group (Figure 5A–C). Core subnetwork analysis identified distinct modules within the CON (Figure 5B) and YC (Figure 5D) groups. A total of 137 significant co-occurrence relationships were detected: 98 in the CON group and 39 in the YC group. Positive correlations were predominant among taxa within the phylum *Pseudomonadota*, whereas negative correlations were observed primarily between *Pseudomonadota* and *Bacillota*; this negative association was particularly evident in the CON group (Figure 5A,B).

### 3.5. Functional Profiles of the Jejunal Microbiome Between the CON and YC Calves

**Figure 6:**
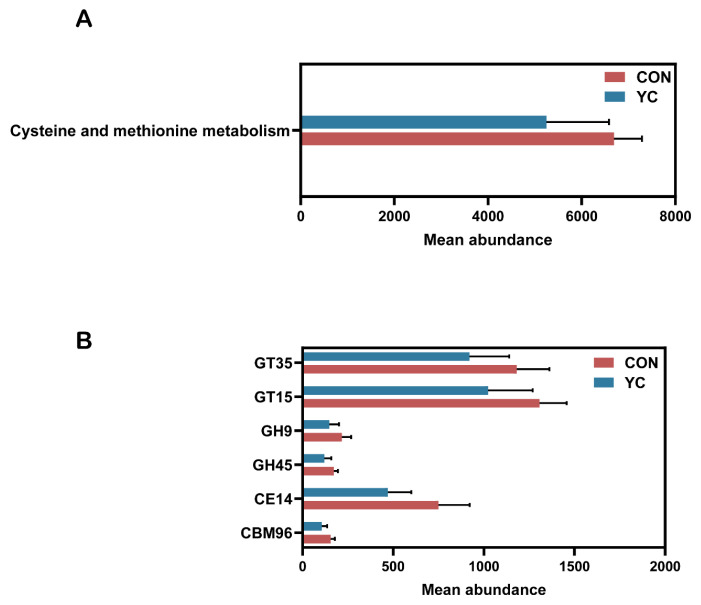
Differences in functional profiles of the jejunal microbiome between CON and YC calves. (**A**) Significantly different KEGG pathways (level 3) between groups. (**B**) Significantly different CAZyme families between groups.

Functional profiling based on the Kyoto Encyclopedia of Genes and Genomes (KEGG) revealed significant differences between groups. At KEGG level 3, the “Cysteine and methionine metabolism” pathway was significantly enriched in the jejunal microbiome of CON calves (*p* < 0.05; Figure 6A).

Annotation against the carbohydrate-active enzymes (CAZymes) database identified a total of 360 CAZyme families. Glycoside hydrolases (GHs; 38.2%) and glycosyltransferases (GTs; 28.8%) were the most abundant categories. Several subfamilies differed significantly between groups, including CE14 (carbohydrate esterases), CBM96 (carbohydrate-binding modules), GH45 and GH9 (glycoside hydrolases), and GT15 and GT35 (glycosyltransferases) (*p* < 0.05; Figure 6B).

### 3.6. Jejunal Metabolome Profiling Differed Between CON and YC Calves

**Figure 7:**
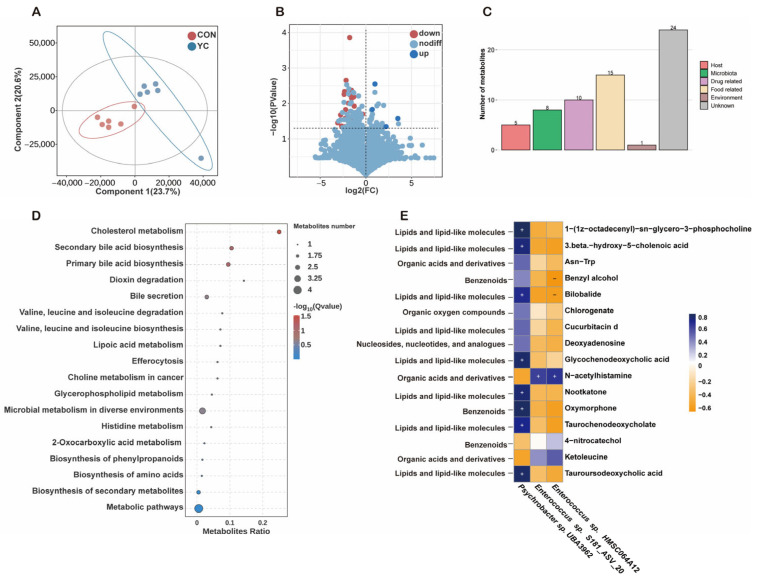
Metabolomic profiles of jejunal digesta in CON and YC calves. (**A**) Partial least squares discriminant analysis (PLS-DA) of the metabolomes of CON and YC calf jejunal digesta. (**B**) Volcano plot of differential metabolites (VIP > 1, *p* < 0.05). (**C**) Identification of metabolites from different sources. (**D**) Metabolic pathway enrichment analysis of differential metabolites. (**E**) Spearman’s rank correlation analysis between differential metabolites and differential microbiota.

Conducted non-targeted metabolomics analysis of the small intestinal microbiome of the CON group and the YC group. Partial least squares discriminant analysis (PLS-DA) revealed distinct metabolic features between the two groups (Figure 7A). A total of 13,142 molecular features were detected across both groups, with 3077 metabolites identified. Using t-test and variable importance in projection (VIP) analysis, 40 metabolites were found to differ significantly between the groups, with 4 metabolites being higher in the YC group and 36 metabolites being higher in the CON group (*p* < 0.05, VIP > 1; Figure 7B).

Specifically, N-acetylhistamine, ketoleucine, 4-nitrocatechol, and fludioxonil were enriched in the YC group. Conversely, metabolites enriched in the CON group included tauroursodeoxycholic acid, taurochenodeoxycholate, serine-conjugated chenodeoxycholic acid, glycochenodeoxycholic acid, oxymorphone, diafenthiuron, kresoxim-methyl acid, nootkatone, Asn-Trp, 2-furancarboxylic acid, cimifugin, alpha-tocopheryl acetate, borrelidin, cucurbitacin D, deoxyadenosine, benzyl alcohol, bilobalide, (Z,E)-tetradeca-9,12-dienol, and 3 beta -hydroxy-5-cholenoic acid. (*p* < 0.05, VIP > 1; Figure 7B).

Pathway enrichment analysis indicated that the most significantly altered metabolic pathways between groups were cholesterol metabolism, secondary bile acid biosynthesis, and primary bile acid biosynthesis (*p* < 0.05; Figure 7D).

Considering the close interaction between the microbiota and the host, we conducted MetOrigin analysis to trace the sources of the differential metabolites. The results showed that 5 metabolites originated from the host, 8 metabolites were derived from the microbiota, and 5 metabolites were shared between both sources (Figure 7C). Further comparative analysis against databases identified 16 significantly different metabolites between the groups. Spearman’s rank correlation analysis was performed between these 16 differential metabolites and microbial taxa. In the YC group, *Enterococcus* sp. *HMSC064A12* and *Enterococcus* sp. *S181_ASV_20* were positively correlated with N-acetylhistamine (*R* > 0.50, *p* < 0.05; Figure 7E), while *Enterococcus* sp. *HMSC064A12* was negatively correlated with benzyl alcohol and bilobalide (*R* < −0.50, *p* < 0.05; Figure 7E). In the CON group, *Psychrobacter* sp. *UBA3962* showed a positive correlation with metabolites such as 1-(1z-octadecenyl)-sn-glycero-3-phosphocholine, 3 beta-hydroxy-5-cholenoic acid, glycochenodeoxycholic acid, taurochenodeoxycholate, and tauroursodeoxycholic acid (*R* < −0.50, *p* < 0.05; Figure 7E).

## 4. Discussion

### 4.1. Study Rationale and Summary of Approach

Heat stress associated with global climate change has become a major environmental constraint on the sustainability of the dairy industry. The prepartum period represents one of the most physiologically challenging stages in a cow’s production cycle; when heat stress occurs during this window, it can synergistically compromise maternal health and performance while also exerting adverse, long-lasting effects on offspring development. Such maternal effects are particularly relevant for the fetal intestinal system, which is undergoing rapid growth and maturation, and may be “programmed” structurally and functionally in ways that increase health risks for neonatal calves. Therefore, developing effective nutritional strategies to mitigate the intergenerational consequences of prepartum heat stress is of considerable importance. Yeast culture (YC), a functional additive rich in bioactive compounds, has demonstrated potential to improve rumen fermentation, strengthen intestinal barrier function, and modulate immune responses in ruminants. However, whether maternal YC supplementation under heat-stress conditions can systemically improve intestinal health in neonatal calves remains unclear.

In the present study, we applied an integrated multi-omics approach to evaluate the offspring effects of maternal YC supplementation during prepartum heat stress. Jejunal structural integrity was assessed by histomorphology, and metagenomic and metabolomic analyses were combined to characterize shifts in the jejunal microbial community and metabolic microenvironment. These complementary datasets provide a systems-level perspective on how maternal YC intervention modulates offspring jejunal function through coordinated changes in intestinal morphology, microbial functional potential, and metabolite profiles.

### 4.2. Maternal YC Supplementation Alleviates Heat Stress and Programs Offspring Systemic Health

Heat stress is a potent inducer of oxidative stress in cattle, typically elevating circulating malondialdehyde (MDA) concentrations compared with thermoneutral conditions [40]. We observed that maternal YC supplementation lowered serum MDA in neonatal calves, a finding consistent with reports that probiotic supplementation enhances antioxidant status and calf health [41]. Concurrently, YC calves exhibited lower serum cortisol concentrations. Cortisol is central to energy metabolism and thermoregulation, and its secretion is upregulated via the hypothalamic–pituitary–adrenal (HPA) axis during heat stress [42,43]. The reduction in cortisol aligns with previous observations in sheep supplemented with probiotics [44] and suggests effective mitigation of the fetal stress response.

In terms of metabolic homeostasis, serum NEFA and BHBA are critical indicators of energy balance, with elevated concentrations signaling metabolic dysregulation [45,46]. The lower NEFA and BHBA concentrations in YC calves indicate that prepartum YC supplementation may program offspring metabolism to maintain homeostasis, thereby reducing the risk of heat stress–induced metabolic disturbances. Regarding immune function, YC calves exhibited greater serum IgA, IgG, and IL-6 concentrations. Serum immunoglobulins are vital for humoral immunity, facilitating pathogen clearance and toxin neutralization [47,48]. Importantly, because cattle possess an epitheliochorial placenta that prevents transplacental immunoglobulin transfer [49], and the calves in this study were sampled before colostrum intake, the elevated IgA and IgG concentrations suggest earlier activation or accelerated maturation of the fetal immune system. This “priming” effect may be attributed to an improved intrauterine environment fostered by maternal YC intervention. The observed increase in IL-6 further supports this hypothesis, consistent with improved growth performance and immune readiness observed in probiotic-supplemented calves [50].

### 4.3. YC Mitigates Heat Stress-Induced Impairment of Offspring Jejunal Morphology

The small intestine is the primary site for nutrient absorption, a process fundamental to neonatal growth and development. Intestinal morphometrics, including VH, CD, and the VH:CD ratio, serve as robust indicators of mucosal health. Specifically, greater VH and an elevated VH:CD ratio are associated with an expanded absorptive surface area and enhanced nutrient transport efficiency [51]. In contrast, heat stress is known to compromise intestinal architecture, typically manifesting as villus atrophy (reduced VH) and crypt hyperplasia (increased CD), thereby lowering the VH:CD ratio and impairing gut function [52].

In our study, jejunal tissue morphology showed that compared to the heat-stressed CON group, calves in the YC group exhibited improved integrity of the intestinal mucosal structure. In the YC group, villi were well-organized and morphologically regular. Although some fragmentations were observed—likely due to mechanical damage during sample preparation—the pathological signs of villus breakage and epithelial shedding were not prominent. In contrast, the CON group showed typical signs of intestinal barrier damage, such as localized villus breakage and disorganized shedding of epithelial cells.

Quantitative analysis revealed that although villus height and crypt depth did not differ individually between groups, the VH:CD ratio was significantly greater in the YC group. This finding is significant given that heat stress typically compromises intestinal morphology; for instance, heat exposure in broilers reduces VH and increases CD [53], and similar reductions in VH are observed in murine models. Consistent with the protective effects observed in our study, Xiao et al. [54] reported that dietary supplementation of yeast fermentation products to neonatal calves reduced CD and increased the VH:CD ratio. Collectively, our results suggest that maternal YC supplementation mitigates the deleterious effects of in utero heat stress on offspring intestinal morphology, potentially through mechanisms of fetal programming.

### 4.4. The Structure of the Small Intestine Microbial Community and the Co-Occurrence Network

Although the “sterile womb” hypothesis has traditionally prevailed, recent detection of microbial DNA in the fetal intestinal contents of ruminants suggests that microbial exposure may commence in utero [55]. However, this concept remains contentious, as low-biomass samples are highly susceptible to contamination, and the presence of microbial DNA does not necessarily equate to viable populations. Notwithstanding the debate regarding the precise timing of initial seeding, the early-life establishment of the gut microbiota is universally recognized as a pivotal factor in programming long-term host health [56].

In the present study, the jejunal microbiome of newborn calves was dominated by *Bacillota*, *Bacteroidota*, and *Pseudomonadota*, a profile consistent with previous characterizations of early-life small intestinal communities in cattle [57,58,59]. *Bacillota* tended to be more abundant in YC calves (although not significantly different). Members of *Bacillota* are important contributors to the fermentation of cellulose-rich substrates and resistant starch, supporting volatile fatty acid production (including acetate and butyrate) and participating in microbial protein synthesis that is relevant for host growth and lactation-related nutrient demands [60,61].

At the species level, *Enterococcus* sp. *HMSC064A12* and *Enterococcus* sp. *S181_ASV_20* were significantly enriched in YC calves. While these specific strains are not yet well characterized, the genus Enterococcus encompasses strains with diverse functional attributes: some serve as effective direct-fed microbials in ruminant production [62,63], whereas others are opportunistic pathogens associated with antimicrobial resistance [64]. The positive association between *Enterococcus* sp. *HMSC064A12* and serum IgG aligns with reports that specific *Enterococcus* strains can stimulate mucosal immune responses [65]. However, this relationship is correlational and does not establish causality; it may reflect direct immunostimulatory effects of the bacterium or a shared upstream response to maternal YC intervention that independently shaped both immune development and microbial ecology. Furthermore, the negative correlation with BHBA implies a potential link between this taxon and improved metabolic stability, although mechanistic validation is required.

In contrast, *Psychrobacter* sp. *UBA3962* was more abundant in CON calves. *Psychrobacter* spp. are Gram-negative, aerobic bacteria within the family *Moraxellaceae*. They have been isolated from foods (e.g., cheese, seafood, and meat) and from animal tissues including the porcine gastrointestinal tract and lamb lungs [66], and have also been reported in the nasal cavity of cattle with respiratory disease [67]. In our dataset, *Psychrobacter* sp. *UBA3962* showed a negative correlation with IgA, which may indicate an association with less favorable mucosal immune status in calves from heat-stressed dams, However, this inference warrants caution; the correlation does not imply causality and may instead reflect a shared response to the adverse intrauterine environment.

Finally, co-occurrence network analysis suggested distinct interaction patterns between groups. The CON network contained more nodes and edges than the YC network, indicating a denser set of inferred microbe-microbe associations under maternal heat stress without YC supplementation. In the YC network, taxa belonging to the phylum *Pseudomonadota* occupied central positions, suggesting that these bacteria may play a pivotal role in community organization. Collectively, these findings support the concept that maternal YC supplementation can reshape early-life jejunal microbial ecology—less by altering global diversity metrics, and more by shifting specific taxa and their inferred interaction structure—thereby potentially influencing the immune and metabolic phenotypes observed in the offspring.

### 4.5. Microbial Functional Adaptations to Stress

Functional profiling revealed that the “cysteine and methionine metabolism” pathway was significantly enriched in the jejunal microbiome of CON calves. Methionine and cysteine are sulfur amino acids (SAA) that possess potent antioxidant properties, attributable to the high reactivity of their sulfur moieties toward reactive oxygen species (ROS). Furthermore, they serve as essential precursors for critical redox regulators and stress-response metabolites, including S-adenosylmethionine, hydrogen sulfide, taurine, and glutathione [68]. Given that methionine is an essential and cysteine a non-essential amino acid for mammals, their combined availability is crucial for maintaining protein synthesis, immune function, and oxidative balance [69]. The enrichment of this pathway in CON calves likely reflects a compensatory microbial response to the heightened oxidative stress environment induced by in utero heat stress, potentially functioning as an adaptive mechanism to bolster antioxidant defenses through increased biosynthesis of SAA and their derivatives.

Methionine serves as a central precursor for succinyl-CoA, homocysteine, cysteine, creatine, and carnitine, while also being essential for polyamine synthesis and phosphatidylcholine metabolism. Furthermore, it drives cellular methylation reactions and facilitates cysteine biosynthesis, thereby sparing the dietary requirement for cysteine [70]. The derived cysteine is utilized not only for protein translation but also for the synthesis of glutathione—a major low-molecular-weight antioxidant—and taurine, a critical osmolyte [71]. Highlighting the significant physiological demand for sulfur amino acids by the gastrointestinal tract, Stoll et al. [72] reported that the net portal balance of methionine accounted for approximately 48% of intake in piglets, indicating substantial intestinal utilization.

Heat stress induces excessive ROS production, and the resulting accumulation can trigger widespread cellular damage, including oxidative injury to DNA and membrane phospholipids, ultimately contributing to cell death, chronic inflammation, and tissue fibrosis [73]. Methionine contributes to antioxidant defense through its metabolic conversion to glutathione, taurine, and other metabolites that modulate immune function [74]. Although studies on methionine restriction suggest that limiting intake can, under certain conditions, enhance glutathione-related responses and alleviate oxidative stress [75]—highlighting the complexity of redox regulation—methionine also functions as a direct ROS scavenger. Its sulfur moiety can be reversibly oxidized to methionine sulfoxide and subsequently repaired by methionine sulfoxide reductases, a cycle that protects proteins from oxidative damage [76]. Collectively, cysteine and methionine metabolism is intrinsically linked to glutathione synthesis and the maintenance of antioxidant capacity.

Under heat-stress conditions, both the host and the gut microbiota experience substantial oxidative stress, leading to the accumulation of reactive oxygen species (ROS). To counteract this burden, microbial communities may upregulate metabolic pathways involved in antioxidant synthesis, including sulfur amino acid metabolism. Methionine supplementation has been shown to alleviate heat stress–induced intestinal injury through improvements in intestinal morphology and tight junction protein expression, enhancement of antioxidant enzyme activities and goblet cell density, and reduction in markers of mucosal damage and lipid peroxidation [77]. These findings indirectly suggest that under conditions of heat stress, methionine is mobilized to support antioxidant defense mechanisms. Furthermore, cysteine—a downstream product of methionine transsulfuration and a critical precursor for glutathione synthesis—is likely recruited to meet the elevated demand for antioxidant capacity [78]. Conversely, the relatively lower activity of this metabolic pathway in the YC group suggests that maternal supplementation may have mitigated reactive oxygen species (ROS) levels within the maternal–fetal unit, possibly by enhancing maternal glutathione status or reducing systemic oxidative stress. Consequently, this alleviation of oxidative burden likely diminished the necessity for the fetus to upregulate compensatory cysteine and methionine metabolic pathways for glutathione generation.

CAZyme profiling further supported the presence of microbial metabolic pressure under maternal heat stress from the perspective of carbon acquisition. In this study, several CAZyme families were significantly upregulated in the CON group compared with the YC group, including glycoside hydrolase (GH) families GH9 and GH45, as well as the carbohydrate esterase (CE) family CE14. GH9 and GH45 are typical endoglucanases that contribute to the degradation of highly crystalline plant cell-wall polysaccharides [79,80]. Carbohydrate esterases cleave ester or amide bonds within carbohydrate substrates, thereby facilitating the deconstruction and remodeling of complex polysaccharides such as chitin, pectin, and hemicellulose [81].

The enrichment of these enzyme families indicates that the jejunal microbiota of CON calves was metabolically restricted, relying on the utilization of recalcitrant structural carbohydrates. A plausible explanation is that heat stress compromised maternal dry matter intake (DMI), which likely established an energy-limited microenvironment, thereby enhancing the utilization of carbon sources by the microorganisms. This consequently restricted the flow of nutrients to the maternal–fetal unit. In contrast, under conditions of adequate nutrition and physiological stability, the microbial community preferentially metabolizes readily fermentable soluble carbohydrates [82]. Therefore, the observed CAZyme distribution is consistent with the adaptive response of the microbiota to carbon limitation and physiological stress. This aligns with general biological principles stating that in nutrient-limited conditions, microorganisms intensify alternative metabolic programs; for instance, bacteria subjected to starvation stress have been shown to upregulate specific stress-adaptation enzymes to ensure survival [83].

### 4.6. YC Modulates the Jejunal Metabolic Microenvironment, Particularly Bile Acid Metabolism

Histamine acetylation is one of the key pathways by which the host inactivates histamine; the product, N-acetylhistamine, is generally considered to have little to no biological activity [84]. In this study, the concentration of N-acetylhistamine in jejunal digesta was significantly lower in CON calves than in YC calves. This pattern may suggest that maternal heat stress suppresses histamine acetylation, thereby limiting normal histamine inactivation and favoring histamine accumulation in the intestinal lumen, which could amplify local inflammatory responses. Conversely, the elevated N-acetylhistamine concentrations observed in YC calves imply a modulation of histamine metabolism toward enhanced inactivation, potentially contributing to the attenuation of intestinal inflammation.

Bile acids (BAs) are a class of steroid acids with distinct physicochemical and biological properties and represent a major component of bile [85]. In mammals, the primary bile acids cholic acid (CA) and chenodeoxycholic acid (CDCA) are synthesized from cholesterol in hepatocytes and secreted into the intestine to facilitate dietary lipid digestion [86]. Bile acids are efficiently reabsorbed from the gut through the enterohepatic circulation, and bile acid receptors are expressed in multiple tissues, including enterocytes, adipose tissue, and liver [87]. Therefore, bile acids function not only as luminal detergents but also as signaling molecules that regulate diverse physiological processes and are often described as hormone-like mediators [88]. Furthermore, BA contribute to mucosal defense mechanisms, and dysregulation of their metabolism has been implicated in the pathophysiology of intestinal disorders, including inflammatory bowel disease [89].

In the CON group, multiple conjugated bile acids were significantly accumulated relative to the YC group. Given that bile acids can traverse the placental barrier and are detectable in amniotic fluid [90], it is plausible that maternal YC supplementation modulated maternal bile acid metabolism, thereby altering fetal exposure in utero. The abnormal accumulation of conjugated bile acids may directly impair mucosal barrier integrity and promote inflammatory responses. Moreover, because most bile acids are reabsorbed in the terminal ileum and returned to the liver via the portal circulation [91], the higher bile acid accumulation observed in the jejunum of CON calves (relative to YC calves) may indicate altered enterohepatic cycling dynamics upstream—potentially reflecting slowed intestinal transit in offspring under maternal heat stress [92]. This interpretation is consistent with our metagenomic evidence showing enhanced amino acid metabolic potential and with the metabolomic finding of elevated ketoleucine, which supports broader metabolic remodeling under heat stress. In agreement, a previous study investigating mechanisms of heat-stress–associated hepatic metabolic disturbance in dairy cows identified candidate metabolite biomarkers, including significantly increased concentrations of leucine (along with urea and creatinine) under heat stress [93]. Ketoleucine is a key intermediate in leucine metabolism and has been used as a functional surrogate in certain contexts due to the low aqueous solubility of leucine [94].

KEGG pathway enrichment analysis revealed significant enrichment in the cholesterol metabolism, primary bile acid biosynthesis, and secondary bile acid biosynthesis pathways, collectively constructing a complete bile acid metabolic regulation axis. This functional profile corroborates the metabolite-level findings, specifically the observed accumulation of conjugated bile acids. The marked changes in cholesterol metabolism suggest that maternal YC supplementation may indirectly affect cholesterol homeostasis in the offspring by modulating the maternal metabolic state. Cholesterol and bile acid metabolism are not isolated pathways but rather part of a liver–gut axis involving both host synthesis and microbial transformation. Cholesterol is not only a crucial component of cell membranes but also a precursor for bile acid synthesis [95,96]. Our study demonstrates that YC intervention impacts both the upstream (cholesterol metabolism, primary bile acid synthesis) and downstream (secondary bile acid synthesis) components of this system, suggesting that the effect of YC is systemic.

Primary bile acids are synthesized in the liver and are converted to secondary bile acids in the intestine through microbial enzymatic activity [97]. This process not only aids in lipid digestion but also plays a critical role in regulating gut microbiota structure, mucosal immunity, and inflammatory responses [98]. According to the Developmental Origins of Health and Disease (DOHaD) theory, changes in the maternal gut microbiota during pregnancy can influence offspring health by altering the microbial composition and metabolite profiles. Heat stress may disrupt the gut microbiota composition and function in both the mother and the offspring, thus disturbing bile acid homeostasis. YC supplementation alleviates maternal heat stress, improving the maternal microbiome structure [99], and consequently alters the metabolite profiles transferred to the fetus via the placenta. Given the concordance between metabolomics findings for bile acid pathways and improvements in small intestinal morphology in the YC group, we propose that maternal YC supplementation may partially promote offspring gut health by modulating the bile acid environment. This likely reflects a transgenerational metabolic regulatory effect of yeast culture along the maternal-offspring axis.

### 4.7. Analysis of Microbiota-Metabolite Associations

The correlation between differential microbiota and differential metabolites in the jejunum indicates that *Enterococcus* species, including *Enterococcus* sp. *HMSC064A12*, are negatively correlated with benzyl alcohol and bilobalide levels. This association suggests that the YC-induced enrichment of *Enterococcus* sp. *HMSC064A12* may contribute to the reduction in these metabolites, thereby promoting intestinal stability. Benzyl alcohol has been shown to damage the integrity of the apical membrane of gastrointestinal cells and vesicles [100], which suggests that its reduced levels may have a protective effect on gut health.

Interestingly, bilobalide has been primarily studied in mouse models and human diseases, where it is reported to alleviate inflammation in osteoarthritis in rats [101]. However, this contrasts with our observation of elevated bilobalide in the jejunum of heat-stressed CON calves. This discrepancy might reflect differences in the metabolic and inflammatory states between species or in response to environmental stress. In contrast, the positive correlation between *Enterococcus* sp. *HMSC064A12*, *Enterococcus* sp. *S181_ASV_20*, and N-acetylhistamine suggests an increased catabolic activity of *Enterococcus* in the gut, leading to enhanced production of N-acetylhistamine. This metabolic conversion is potentially beneficial, as it limits the accumulation of bioactive histamine and may thereby mitigate heat-stress–induced intestinal damage.

### 4.8. Limitations and Future Perspectives

It should be noted that while rapid postnatal sampling was employed to minimize environmental exposure, the potential contribution of microbes acquired during the very act of birth to the earliest jejunal community cannot be entirely ruled out. Nonetheless, the observed significant differences between CON and YC groups are likely driven by the maternally programmed differences in the fetal gut environment and the initial microbial inoculum.

This study has certain limitations. Firstly, only male calves were used. Although this enhanced internal validity by controlling for gender-related variations, it implies that the research results may not be directly applicable to female calves. Future studies should include both male and female calves to confirm whether the beneficial effects of maternal supplementation of YC are consistent across different genders.

## 5. Conclusions

In conclusion, supplementing maternal diets with yeast culture (YC) alleviated heat stress-induced increases in rectal temperature in dairy cows and enhanced jejunal development in offspring calves through intrauterine programming. YC elevated serum levels of IgA and IgG in calves, reduced markers of oxidative and metabolic stress, improved jejunal villus architecture and microbial composition, enriched beneficial intestinal cocci, and modulated bile acid metabolism pathways. These collective changes strengthened intestinal barrier function and immune status in calves; however, the long-term regulatory mechanisms underlying YC’s effects on offspring health remain to be further elucidated.

## Data Availability

The original contributions presented in this study are included in the article. Further inquiries can be directed to the corresponding author.

## Abbreviations

The following abbreviations are used in this manuscript:

BCS	Body condition score
BHBA	Beta-hydroxybutyrate
CAZymes	Carbohydrate-active enzymes
CD	Crypt depth
FDR	False discovery rate
GHs	Glycoside hydrolases
GTs	Glycosyltransferases
H&E	Hematoxylin and eosin
HS	Heat stress
KEGG	Kyoto Encyclopedia of Genes and Genomes
LCA	Lowest common ancestor
NEFA	Nonesterified fatty acids
OPLS-DA	Orthogonal partial least squares discriminant analysis
PLS-DA	Partial least squares discriminant analysis
ROS	Reactive oxygen species
SAAs	Sulfur amino acids
THI	Temperature–humidity index
TMR	Total mixed ration
VH	Villus height
VH:CD	Villus height to crypt depth
VIP	Variable importance in projection
YC	Yeast culture

## Appendix A

**Table A1:** Ration composition and nutrient levels (dry matter basis, %).

Ingredient	Content	Nutrient Levels	Content
Corn silage	31.92	CP	14.11
Oat Hay	26.98	EE	4.83
Beet particles	8.29	NDF	33.87
Choline	0.08	ADF	21.62
Corn Meal	5.44	Starch	23.96
Bean pulp	8.46	Ca	1.20
DDGS	9.46	P	0.35
Cottonseed	2.63		
Canola Meal	2.46		
Corn gluten feed	2.64		
Premix	1.64		
Total	100.0		

Each kilogram of premix contains vitamin A 3500 kIU, vitamin D 1000 kIU, vitamin E 65 kIU, copper 6000 mg, manganese 15,000 mg, zinc 11,500 mg, selenium 100 mg, iodine 250 mg, and cobalt 300 mg.

**Table A2:** Average Temperature and Humidity Chart of the Cowshed.

Time	Average Temperature (°C)	Average Relative Humidity (%)	Average Temperature and Humidity Index (THI)
7:00	27.1	89	79.5
14:00	32.8	67.6	85
22:00	27.8	82.2	79.6

**Table A3:** Summary of sequence data generated from.

Sample	Raw Reads	Optimized Reads	Contigs	N50
CON-1	69,484,520	68,173,920	61,207	5863
CON-2	65,490,640	64,237,962		
CON-3	68,557,194	67,213,112		
CON-4	61,767,890	60,629,348		
CON-5	68,777,206	67,300,588		
CON-6	63,602,106	62,282,122		
YC-1	81,394,914	79,614,124	64,027	4550
YC-2	67,869,606	66,570,658		
YC-3	66,994,116	65,338,866		
YC-4	67,572,548	66,063,302		
YC-5	81,857,842	79,692,548		
YC-6	63,083,808	61,584,144		
Total	826,452,390	808,700,694	125,234	10,413
Mean	68,871,033	67,391,725	62,617	5207
SEM	1,857,286	1,791,213	1410	657

CON: heat stress group; YC: yeast culture group; SEM: standard error of the mean.
